# Bounding tractogram redundancy

**DOI:** 10.3389/fnins.2024.1403804

**Published:** 2024-07-23

**Authors:** Sanna Persson, Rodrigo Moreno

**Affiliations:** ^1^Department of Biomedical Engineering and Health Systems, KTH Royal Institute of Technology, Huddinge, Sweden; ^2^MedTechLabs, BioClinicum, Karolinska University Hospital, Solna, Sweden

**Keywords:** diffusion MRI, tractography, tractogram filtering, tractogram redundancy, Hoeffding's inequality, Bayesian estimation

## Abstract

**Introduction:**

In tractography, redundancy poses a significant challenge, often resulting in tractograms that include anatomically implausible streamlines or those that fail to represent the brain's white matter architecture accurately. Current filtering methods aim to refine tractograms by addressing these issues, but they lack a unified measure of redundancy and can be computationally demanding.

**Methods:**

We propose a novel framework to quantify tractogram redundancy based on filtering tractogram subsets without endorsing a specific filtering algorithm. Our approach defines redundancy based on the anatomical plausibility and diffusion signal representation of streamlines, establishing both lower and upper bounds for the number of false-positive streamlines and the tractogram redundancy.

**Results:**

We applied this framework to tractograms from the Human Connectome Project, using geometrical plausibility and statistical methods informed by the streamlined attributes and ensemble consensus. Our results establish bounds for the tractogram redundancy and the false-discovery rate of the tractograms.

**Conclusion:**

This study advances the understanding of tractogram redundancy and supports the refinement of tractography methods. Future research will focus on further validating the proposed framework and exploring tractogram compression possibilities.

## 1 Introduction

Diffusion-weighted magnetic resonance imaging (DW-MRI) has emerged as a revolutionary tool for non-invasively probing the complex architecture of white matter tracts in the living brain. The technique captures the diffusion of water molecules, which preferentially occurs along the length of axonal fibers, thereby providing insights into the orientation and integrity of neural pathways. Tractography algorithms leverage this information to reconstruct the three-dimensional trajectories of white matter tracts, known as streamlines, resulting in a tractogram which is a comprehensive map of neural connections within the brain (De Benedictis et al., 2016; Hau et al., 2017; Maffei et al., 2018; Jeurissen et al., 2019; Henderson et al., 2020). Applications range from connectivity network studies (Yeh et al., 2021; Zhang et al., 2022), segmentation (Wasserthal et al., 2018; Rheault et al., 2020, 2022; Warrington et al., 2020; Bertò et al., 2021; Maffei et al., 2021; Schilling et al., 2021; Siegbahn et al., 2022), to the identification of neural pathways for surgery planning (Henderson et al., 2020; Yang et al., 2021).

Many tractography algorithms have been proposed in the last two decades using diverse methodologies (Mori et al., 1999; Basser et al., 2000; Smith et al., 2012; Christiaens et al., 2015; Neher et al., 2017; Poulin et al., 2017, 2019; Konopleva et al., 2018; Jeurissen et al., 2019; Théberge et al., 2021; Sinzinger and Moreno, 2022; Legarreta et al., 2023). This large number of available methods has made it difficult for end users to choose the most appropriate tractography algorithm for their applications. Furthermore, there is little consensus on which parameters to use, such as the most appropriate number of streamlines. In order to address this issue, one of the goals of the International Society for Magnetic Resonance in Medicine (ISMRM) 2015 Tractography Challenge (Maier-Hein et al., 2017) was to help end users by quantitatively assessing the performance of tractography pipelines in a realistic phantom. This challenge used the Tractometer (Côté et al., 2013) for this aim. The Tractometer uses regions of interest (ROIs) to define six different measurements. While using ROIs gives insights into the quality of the tractogram, such a methodology is unable to assess the quality of individual streamlines. For example, anatomically implausible streamlines that do not go outside of a bundle segmentation mask connecting two brain regions will not penalize Tractometer measurements. This restriction can potentially affect connectivity and tractometry analyses (Chandio et al., 2020). Thus, there is currently an unmet need to create new measurements that can address the limitations of the Tractometer. This study contributes to this goal by leveraging tractogram filtering methods.

Despite its widespread application in neuroscience research and clinical settings, tractography faces significant challenges (Daducci et al., 2016; Maier-Hein et al., 2017; Schilling et al., 2019). One of the most critical issues is the presence of false-positive streamlines within tractograms (Daducci et al., 2016; Jörgens et al., 2021). False-positive streamlines manifest as either anatomically implausible streamlines that do not correspond to true neural pathways or as overlapping with other streamlines (duplicates) that result in redundancy in the representation of the diffusion signal. We refer to the duplicated streamlines as redundant. These erroneous streamlines can obscure the true structural connectivity, leading to misinterpretations in both research and clinical applications (Garyfallidis et al., 2012; Durantel et al., 2022).

This study aims to create a statistical framework to estimate the lower and upper bounds of tractogram redundancy from per-streamline estimates obtained with tractogram filtering. These estimates can potentially be used to rank tractography pipelines by their inefficiency, with the ambition of fostering research for improved tractography methods. Although the proposed framework is generic, we use three specific tractogram filtering methods: ExTractor (Petit et al., 2023), randomized spherical-deconvolution-informed filtering of tractograms (rSIFT) (Hain et al., 2023), and randomized convex optimization modeling for microstructure informed tractography (rCOMMIT) (Wan, 2023).^1^

## 2 Background

### 2.1 Redundancy in tractograms

A *false-positive* streamline in a tractogram does not contribute to, or may even detract from, the accurate representation of the brain's white matter architecture as inferred from diffusion MRI data. False positives can manifest either through streamlines that do not correspond to anatomically plausible structures or redundant streamlines that do not enhance the fidelity of the tractogram to the diffusion signal (duplicates), thereby failing to improve or clarify the depiction of the brain's structural connectivity. The fraction of duplicated streamlines is also referred to as the redundancy in the tractogram and constitutes an important distinction from the total number of false-positive streamlines. An anatomically implausible streamline would not be seen in a brain and is likely the result of an error produced during tractography. The identification and removal of these streamlines aim to refine the tractogram, ensuring that it more faithfully reflects the underlying neural pathways and microstructural characteristics.

### 2.2 Tractogram filtering methods

Tractography filtering is the process of refining a tractogram by identifying and removing streamlines that are considered false positives. This process is essential to enhance the quality and usability of tractograms for both research and clinical applications. Filtering methods vary in their approach, with some focusing on the anatomical plausibility of streamlines, while others aim to ensure that the streamline distribution corresponds to the underlying diffusion signal (Jörgens et al., 2021).

The concept of tractography filtering is rooted in the understanding that not all streamlines in a tractogram contribute equally to the representation of the white matter structure. Some streamlines may be artifacts of the tractography process, while others may represent genuine neural pathways but are overrepresented due to biases in the algorithm. Filtering methods aim to identify these discrepancies and adjust the tractogram accordingly to produce a more accurate and reliable representation of the brain's white matter. Specifically, in this study, we used three different tractogram filtering methods: ExTractor, rSIFT, and rCOMMIT.

#### 2.2.1 ExTractor: filtering for anatomical plausibility

ExTractor (Petit et al., 2023) is a rule-based automatic pipeline designed to enhance the anatomical plausibility of tractograms by filtering streamlines inconsistent with known neuroanatomical principles. ExTractor operates on the premise that every cortical area is interconnected with other cortical and subcortical regions via association, commissural, and projection fibers, which adhere to a certain anatomical organization. The method is grounded in the neuroanatomical categorization established by previous research (Meynert, 1885; Ludwig and Klingler, 1956; Crosby, 1963; Schmahmann and Pandya, 2006; Nieuwenhuys et al., 2008).

In the process of automatic filtering, ExTractorFlow (Cousineau et al., 2017; Di Tommaso et al., 2017; Kurtzer et al., 2017), an implementation of the ExTractor method, employs anatomical rules derived from the structural organization of white matter fibers. The filtering method uses ROIs from established brain templates (Oishi et al., 2009) to enforce sequential filtering conditions that discard streamlines unlikely to represent true anatomical pathways. For instance, streamlines are considered implausible if they are shorter than a specified length, make excessive loops, terminate along ventricular surfaces, or end within deep white matter structures inconsistent with expected tract trajectories.

#### 2.2.2 SIFT: spherical-deconvolution-informed filtering of tractograms

SIFT (Smith et al., 2013) refines tractograms by selectively discarding streamlines that poorly fit the fiber orientation distributions (FODs) derived from constrained spherical deconvolution (Tournier et al., 2007). It operates under the premise that the streamline density within a voxel should be proportional to the FOD amplitude, which reflects the volume of white matter fibers in that orientation. Streamlines are assessed based on their contribution to the FODs, and those that over-represent or under-represent the FOD-derived fiber volume are filtered out. This targeted removal reduces reconstruction biases, such as overemphasis on longer pathways or straighter courses in branching tracts, leading to tractograms that more plausibly represent the structural connectivity. SIFT works independently of the tractography method.

In SIFT, each streamline is evaluated for its alignment with the FODs, which provide a probabilistic estimate of the direction and density of fibers within each voxel. SIFT employs a cost function that quantifies the discrepancy between the streamline density and the FOD amplitude across the tractogram. Streamlines contributing to an excess of density in comparison to the FODs are deemed false-positive and are preferentially removed, while those in deficit areas are retained, ensuring a balance that mirrors the estimated fiber volumes. SIFT does not distinguish between redundant streamlines and anatomically implausible ones.

The filtering process is iterative, with a gradient descent approach guiding the selection of streamlines for removal. The algorithm calculates a proportionality coefficient, which scales the streamline density to the FOD amplitude, and it adjusts this coefficient dynamically as streamlines are removed. This ensures that the remaining streamline distribution continues to provide the best possible fit to the FODs throughout the filtering process.

#### 2.2.3 COMMIT: convex optimization modeling for microstructure-informed tractography

COMMIT (Daducci et al., 2015) is a filtering algorithm that refines tractograms by leveraging a convex optimization framework to incorporate microstructural tissue properties, discerning between anatomically plausible tracts and artifacts. It adjusts the weight of each candidate fiber derived from standard tractography to best fit the diffusion signal to ensure the quantitative integrity of the tractogram. COMMIT models the diffusion signal within each voxel as a linear combination of the diffusion responses from these tracts. The method applies convex optimization to solve for the global weights of these tracts, effectively pruning the tractogram by removing or down-weighting contributions that do not align with the observed diffusion signal. This results in a filtered tractogram that more accurately reflects the underlying structural connectivity with reduced redundancy and improved anatomical plausibility.

#### 2.2.4 Randomized SIFT and COMMIT filtering algorithms

One issue with both SIFT and COMMIT is that they cannot be used for estimating the anatomical plausibility of individual streamlines. Indeed, the very same streamline can be accepted or rejected depending on the composition of the tractogram. This is because both methods aim to reject both anatomically implausible and redundant streamlines. Thus, an anatomically plausible streamline can be rejected if it is deemed a duplicate. This issue has been addressed by randomized SIFT (rSIFT) and COMMIT (rCOMMIT). rSIFT (Hain et al., 2023) introduces a sampling method offering a statistical framework for the evaluation of each streamline's inherent importance to the tractogram. By employing random sub-sampling, rSIFT iteratively applies the SIFT algorithm across numerous tractogram subsets, effectively creating a distribution of filtering outcomes for each streamline. This process enables the quantification of streamline acceptance rates, which serve as a probabilistic measure of the streamline's fidelity to the underlying diffusion signal.

rSIFT uses the collective behavior of streamlined subsets to infer the likelihood of anatomical plausibility. The method uses the variability introduced by the randomization process to discern between duplicates and outliers that are inconsistent with the diffusion data. This distinction is critical, as it addresses the intrinsic limitations of global optimization strategies in conventional SIFT, which may penalize both types of streamlines. rCOMMIT (Wan, 2023) (see text footnote ^1^) uses the same sampling and voting method as rSIFT but implements COMMIT as the filtering method of the tractograms. That is, each subset is filtered by weights that are larger than zero.

The unavoidable result of both randomized algorithms is that the aggregation over many tractogram subsets is prohibitively computationally expensive. Efforts have been made to imitate the filtering algorithm with deep learning using streamline-by-streamline classification. The current accuracy of those methods is in the range of 80%. In order to obtain an accurate measurement of redundancy, we used the standard rSIFT and rCOMMIT in the experiments.

## 3 Methods

### 3.1 Quantifying tractogram redundancy

In Section 2.1, we propose a definition of redundancy that distinguishes between the total number of false-positive streamlines and the number of duplicates. We now attempt to formalize this notion of redundancy further in a tractogram. Assume there is a tractogram T^*^, which is the optimal representation of the underlying structural connectivity. In line with previous research, we assume for the sake of simplicity that the unfiltered tractogram *T* is redundant, i.e., T^*^⊆*T*. In particular, we assume that


$$\begin{array}{l} |T|=|T^{*}|+|D|+|I|, \end{array}$$

where |*D*| and |*I*| are the number of redundant (duplicated) and anatomically implausible streamlines, respectively. We aim to propose a framework for bounding the fraction of false-positive and redundant streamlines in tractograms:


$$\begin{array}{l} l\leq \frac{\left|T|-|T^{*}\right|}{\left|T\right|}\leq u, \end{array}$$

where *l* and *u* are the lower and upper bounds of the fraction of false-positive streamlines, given by


$$\begin{array}{l} l\equiv \frac{\left|I\right|}{\left|T\right|}, \end{array}$$

$$\begin{array}{l} u\equiv \frac{\left|I|+|D\right|}{\left|T\right|}. \end{array}$$

The fraction of false-positive streamlines can also be referred to as the false discovery rate (FDR), which is the fraction of streamlines in the tractogram that are falsely discovered


$$\begin{array}{c} \text{FDR}=\frac{FP}{FP\;+\;TP}=\\ \frac{\text{false positive streamlines}}{\text{false positive streamlines (filtered) }+\;\text{true positive streamlines}} \end{array}$$

It should be noted that, depending on the application, one of the bounds is more relevant. For example, duplicates are not an issue for bundle segmentation. Thus, *l* can be used as a measure of the FDR. In turn, structural connectivity analyses and bundle-wise tractometry can be affected by redundancy, so *u* can be used instead. That is, we bound the redundancy *R* as 0 ≤ *R* ≤ *u*−*l*.

As described in Jörgens et al. (2023), some tractogram filtering methods restrictively filter only the anatomically implausible streamlines, while others filter both implausible and redundant ones. The former can be used to estimate the lower bound *l*, while the latter is useful for *u*. In our case, ExTractor is appropriate for *l* and rSIFT and rCOMMIT for *u*, as described in the following subsections. Both rSIFT and rCOMMIT use streamline attributes and the consensus of filtering different subsets to assess streamline plausibility.

### 3.2 Estimating the lower bound with ExTractor

The lower bound of the FDR requires a filtering method focused on the anatomical plausibility of streamlines. It should be noted that the definition of anatomical plausibility is not unambiguous, therefore we consider the notion of geometrical plausibility as a surrogate that allows us to quantify the lower bound *l*. ExTractor is a method that can be used for this aim since it does not discard redundant streamlines, which is necessary to estimate the lower bound. In particular, we estimated the FDR lower bound as the percentage of rejected streamlines with ExTractor.

It is important to note that, unlike SIFT and COMMIT, the filtering decision of ExTractor on every streamline does not depend on the composition of the tractogram. The main implication of this is that ExTractor will not benefit from randomized approaches to estimate acceptance rates, as is the case with SIFT and COMMIT. Thus, ExTractor is applied only once per tractogram.

### 3.3 Estimating the upper bound

SIFT and COMMIT, and consequently, rSIFT and rCOMMIT, target both anatomically implausible and redundant streamlines. Thus, combinations of rSIFT and rCOMMIT are good candidates for estimating the upper bound of the streamline FDR. We estimated the upper bound using two methodologies, as described below.

#### 3.3.1 Upper bound by sub-sampling with Hoeffding's bound

In the methods of rSIFT and rCOMMIT, tractogram filtering is repeated over randomized samples from the original tractogram without replacement. This property allows us to compute a probabilistic bound for the deviation of the average FDR from the expected value.

Assume that the tractogram filtering method has been applied to *m* subsets. Let *X*_*i*_ be the random variable representing the number of false-positive streamlines in the *i*-th subset, *A*_*i*_ of size *n*_*i*_. Since each streamline in a subset can either be classified as false-positive or not, we have that *X*_*i*_ is bounded. Specifically, 0 ≤ *X*_*i*_ ≤ *n*_*i*_, where *n*_*i*_ is the total number of streamlines in subset *A*_*i*_.

Let *S*_*m*_ = *X*_1_ + ⋯ + *X*_*m*_ be the total number of false-positive streamlines across all subsets. The expected value of *S*_*m*_ is given by


$$\begin{array}{l} \text{E}\left[S_{m}\right]=\overset{m}{\underset{i=1}{\sum }}\text{E}\left[X_{i}\right]. \end{array}$$

Applying Hoeffding (1963)'s theorem to the sum *S*_*m*_, we can bound the probability that the observed total number of false-positive streamlines deviates from its expected value by at least a certain amount *t* > 0. Specifically, for all *t* > 0,


(1) $$\begin{array}{l} \text{P}\left(|S_{m}-\text{E}\left[S_{m}\right]|\geq t\right)\leq 2\exp\left(-\frac{2t^{2}}{\overset{m}{\underset{i=1}{\sum }}n_{i}^{2}}\right)=p, \end{array}$$

where *p* = 0.05 provides a *t* that gives a 95% confidence interval around *S*_*m*_, that is given by


$$\begin{array}{l} t=\sqrt{-\frac{\overset{m}{\underset{i=1}{\sum }}n_{i}^{2}}{2}\log\left(\frac{p}{2}\right)}. \end{array}$$

This inequality provides a probabilistic upper bound on the deviation of the observed number of false-positives from the expected value given by *S*_*m*_+*t*. For ease of interpretation, we present this bound normalized as


(2) $$\begin{array}{l} u_{Hoeff}=\frac{S_{m}+t}{\overset{m}{\underset{i=1}{\sum }}n_{i}}. \end{array}$$

If the subsets are of equal size, i.e., *n*_*i*_ = *n* for all *i*, then the bound simplifies to


$$\begin{array}{l} \text{P}\left(|S_{m}-mrn|\geq t\right)\leq 2\exp\left(-\frac{2t^{2}}{mn^{2}}\right). \end{array}$$

This setting is useful for estimating upper bounds for specific sampling sizes, as done in rSIFT and rCOMMIT.

By choosing an appropriate value of *t*, we can make statements about the confidence with which the observed FDR does not exceed the expected streamlined FDR by more than the specified amount. For example, setting *t* = ϵ*mn*, where ϵ represents the acceptable deviation from the expected proportion of falsely discovered streamlines on a per-streamline basis, we obtain


$$\begin{array}{l} \text{P}\left(|\frac{S_{m}}{mn}-r|\geq \epsilon \right)\leq 2\exp\left(-2m\epsilon^{2}\right), \end{array}$$

where *r* is the expected FDR in a subset. This result can be used to determine the number of subsets *m* necessary to achieve the desired confidence level for bounding the FDR.

For the upper bound of the FDR, we use the one-sided bound of Equation 1 given by:


$$\begin{array}{l} \text{P}\left(S_{m}-\text{E}\left[S_{m}\right]\geq t\right)\leq \exp\left(-\frac{2t^{2}}{\overset{m}{\underset{i=1}{\sum }}n_{i}^{2}}\right), \end{array}$$

where *t* is any real number.

#### 3.3.2 Upper bound with an empirical Bayesian approach

Using an empirical Bayesian approach, we can also bound the streamlined FDR in tractograms by considering the acceptance rates obtained through randomized tractography filtering algorithms. We first establish an empirical prior based on the observed data, then compute the likelihood for each streamline, update to form the posterior probability, and aggregate the results to provide an upper bound for the FDR.

Given *N* streamlines and *m* subsets, let *a*_*i*_ denote the acceptance rate of the *i*-th streamline, which is the proportion of subsets where the streamline is classified as a true positive. We model the prior distribution of acceptance rates using a Beta distribution, whose parameters α and β are estimated by:


$$\begin{array}{l} \alpha =\bar{a}\left(\frac{\bar{a}\left(1-\bar{a}\right)}{s^{2}}-1\right),\;\beta =\left(1-\bar{a}\right)\left(\frac{\bar{a}\left(1-\bar{a}\right)}{s^{2}}-1\right), \end{array}$$

where $\bar{a}$ and *s*^2^ are the sample mean and variance of the acceptance rates, respectively. The likelihood of observing the acceptance rate *a*_*i*_ for the *i*-th streamline, assuming a binomial model, is given by:


$$L(a_{i};k_{i},v_{i})=\left(\begin{array}{c} v_{i}\\ k_{i} \end{array}\right)a_{i}^{k_{i}}{\left(1-a_{i}\right)}^{v_{i}-k_{i}},$$

where *k*_*i*_ is the number of accepted classifications and *v*_*i*_ is the total number of subsets in which streamline *i* appears. The posterior distribution for each streamline is under the Beta-binomial conjugacy, also a Beta distribution


$$\begin{array}{l} {\text{P}}_{i}=\text{Beta }\left(\alpha +k_{i},\beta +v_{i}-k_{i}\right). \end{array}$$

To aggregate the posteriors, we compute the mean and variance of the posterior probabilities of the FDR across all streamlines:


$$\begin{array}{l} \bar{\text{FDR}}=1-\frac{1}{N}\overset{N}{\underset{i=1}{\sum }}\frac{\alpha +k_{i}}{\alpha +k_{i}+\beta +v_{i}-k_{i}}, \end{array}$$

where *N* is the total number of streamlines.

To describe the variance of the posterior probabilities for the FDR across all streamlines, we must consider not only the individual variances of each posterior but also the covariance among them. The total variance of the mean of the posterior probabilities can be expressed as:


$$\begin{array}{l} \sigma_{\bar{\text{P}}}^{2}=\frac{1}{N^{2}}\left(\overset{N}{\underset{i=1}{\sum }}\sigma_{{\text{P}}_{i}}^{2}+\underset{i\neq j}{\sum }\text{cov}\left({\text{P}}_{i},{\text{P}}_{j}\right)\right). \end{array}$$

Given the high dimensionality of most tractograms, calculating the full covariance matrix between all pairs of streamlines is computationally prohibitive. To address this challenge, we can estimate an upper bound on the variance of the mean posterior probability by assuming the maximum possible variance from the individual posteriors. This approach circumvents the need for explicit covariance terms, instead employing the aggregate effect of the maximum variance among the individual probabilities. Consequently, we define our conservative upper bound on the variance as:


$$\begin{array}{l} \sigma_{\bar{\text{P}},\text{upper}}^{2}=\frac{1}{N^{2}}{\left(\overset{N}{\underset{i=1}{\sum }}\sqrt{\sigma_{{\text{P}}_{i}}^{2}}\right)}^{2} \end{array}$$

This upper bound effectively assumes perfect positive correlation among streamlines, thereby reflecting the maximal potential covariance and providing a conservative estimate of variability. Due to the high dimensionality of tractograms, often in the order of millions of streamlines, the central limit theorem ascertains that the distribution of the entire tractogram FDR will be normally distributed. Subsequently, the upper 95% confidence bound on the mean posterior probability of the FDR is computed as:


$$\begin{array}{l} u_{Bayes}=\bar{\text{FDR}}+Z_{0.95}\sqrt{\sigma_{\bar{\text{P}},\text{upper}}^{2}} \end{array}$$

where *Z*_0.95_ represents the 95th percentile of the standard normal distribution. This Bayesian approach provides a conservative estimate of the FDR in the tractogram even when the covariance is not directly computable.

### 3.4 Estimators of streamline probabilities

The presented methods to estimate upper bounds require estimates of streamline FDR in different subsets. For this, we measure FDR as 1—the acceptance rate of rSIFT or rCOMMIT. A streamline that has a high acceptance rate can also be considered non-redundant. In addition, an alternative is combining rSIFT and rCOMMIT acceptance scores to estimate FDR.

#### 3.4.1 Intersection between rSIFT and rCOMMIT

In this estimator, we compute a filtering result based on the computed acceptance probabilities for both rSIFT and rCOMMIT. We obtain the corresponding filtering result by setting a threshold θ, such that a streamline is considered non-redundant if its acceptance probability exceeds this threshold in both methods. Formally, for a given streamline *i*, let *a*_*i*_rSIFT__ and *a*_*i*_rCOMMIT__ denote its acceptance probabilities according to rSIFT and rCOMMIT, respectively. The streamline is included in the filtered tractogram if *a*_*i*_rSIFT__>θ and *a*_*i*_rCOMMIT__>θ. The estimator is then defined as:


$$\begin{array}{l} {\hat{\text{P}}}_{i}^{\text{int}}\left(\theta \right)=I\left(a_{i_{\text{rSIFT}}}>\theta \right)\cdot I\left(a_{i_{\text{rCOMMIT}}}>\theta \right), \end{array}$$

where ${\hat{\text{P}}}_{i}^{\text{int}}$ is the acceptance probability of the intersection of rSIFT and rCOMMIT for a specific threshold θ and *I* is the indicator function. The choice of θ can be based on the desired specificity and sensitivity trade-off, and it can be adjusted according to the distribution of acceptance probabilities. This method provides a straightforward way to combine information from both methods and requires less data in the different subset constitutions than the following methods.

#### 3.4.2 Minimal acceptance rate on a streamlined basis

One issue with considering the intersection of rSIFT and rCOMMIT is that we need to set a specific threshold θ, which can be difficult to choose. An alternative to this is to estimate the streamline's probability of being non-redundant by considering the minimum of the normalized acceptance counts across different methods. Specifically, for each streamline, we look at the number of times it has been accepted by both the rSIFT and rCOMMIT algorithms, normalized by the number of occurrences of that streamline in the respective method's subsets. This method creates a “pseudo-subset” where the streamline's acceptance is evaluated based on its most conservative acceptance rate across the methods for each subset size.

For a given streamline *i*, let *k*_*i*_rSIFT__ be the number of times streamline *i* is accepted by rSIFT, and *v*_*i*_rSIFT__ be the number of subsets in which streamline *i* appears according to rSIFT. Similarly, let *k*_*i*_rCOMMIT__ and *v*_*i*_rCOMMIT__ denote the corresponding counts for rCOMMIT. The maximal valid filtering estimator is then defined as the minimum of the normalized acceptance rates across the methods for each subset size


$$\begin{array}{l} k_{ij},\;v_{ij}=\underset{j}{\mathrm{argmin}}\left(\frac{k_{i,\;\text{rSIFT}}}{v_{i,\;\text{rSIFT}}},\frac{k_{i,\;\text{rCOMMIT}}}{v_{i,\;\text{rCOMMIT}}}\right), \end{array}$$

with


$$\begin{array}{l} {\hat{\text{P}}}_{i}^{\text{min}}=\min\left(\frac{k_{i,\;\text{rSIFT}}}{v_{i,\;\text{rSIFT}}},\frac{k_{i,\;\text{rCOMMIT}}}{v_{i,\;\text{rCOMMIT}}}\right), \end{array}$$

where ${\hat{\text{P}}}_{i}^{\text{min}}$ is the minimum acceptance probability of *i*, and *j* is the tractography filtering method (rSIFT or rCOMMIT). This estimator considers each streamline's relative acceptance rate, providing a conservative estimate of its probability of being non-redundant. It is particularly useful when one wishes to ensure that a streamline is consistently accepted across multiple filtering methods before considering it non-redundant.

#### 3.4.3 Pooled acceptance rate

In this approach, we pool the subsets from both rSIFT and rCOMMIT to create a set of meta-subsets. The pooling process involves combining the subsets from each method, thereby increasing each streamline's total number of observations. Given that both methods are assumed to provide valid filtering results, their combination is expected to enhance the stability of the acceptance rate estimation due to the increased number of samples while reducing bias toward any specific tractography filtering method.

For each streamline *i*, the pooled acceptance probability ${\hat{\text{P}}}_{i}^{\text{pooled}}$ is calculated based on its acceptance across all meta-subsets. If *k*_*i*, rSIFT_ and *k*_*i*, rCOMMIT_ represent the number of times streamline *i* is accepted in rSIFT and rCOMMIT subsets, respectively, and *n*_rSIFT_ and *n*_rCOMMIT_ are the total numbers of subsets for each method, the pooled estimator is then:


$$\begin{array}{l} {\hat{\text{P}}}_{i}^{\text{pooled}}=\frac{k_{i,\;\text{rSIFT}}+k_{i,\;\text{rCOMMIT}}}{n_{\text{rSIFT}}+n_{\text{rCOMMIT}}}. \end{array}$$

This estimator reflects the overall acceptance of a streamline across the combined evidence from both filtering methods.

### 3.5 Data

We use a subset of the Human Connectome Project that consists of seven subjects from a dataset pre-processed by Glasser et al. (2013) with tractograms generated by Wasserthal et al. (2018) using the iFOD2 method as developed by Tournier et al. (2010). Each tractogram consists of 10 million streamlines with a range of 40–250 mm in length, was generated with anatomically constrained tractography with a step size of 0.625 mm, and covers the entire white matter volume. The subset of HCP subjects was also used by rSIFT (Hain et al., 2023) and rCOMMIT (Wan, 2023). The streamlines have been compressed to their most significant points with the method developed by Presseau et al. (2015) using a tolerance level of 0.35mm. The rSIFT parameters are the same as in Hain et al. (2023). For the method of rCOMMIT, we randomly sample tractogram subsets without replacement and run the COMMIT algorithm with the Stick-Zeppelin-Ball model. The parameters used were: axial diffusivity of 1.7 × 10^−3^, perpendicular diffusivity of 0.51 × 10^−3^, isotropic diffusivities of 1.7 × 10^−3^ and 3 × 10^−3^ with a tolerance of 1 × 10^−3^, and maximum iterations of 1,000. Table 1 reports the subset sizes and number of subsets used in the experiments.

**Table 1:** Subset sizes (in thousands) and number of subsets per subset size used for computing rSIFT and rCOMMIT.

Subset sizes	250	500	1,250	2,500	5,000	10,000
Number of subsets	200	100	40	20	10	5

## 4 Experimental results

### 4.1 Lower bound estimation with ExTractor

The lower bound of the FDR was computed with the ExTractor algorithm (Petit et al., 2023) with implementation in Singularity and NextFlow (Cousineau et al., 2017; Di Tommaso et al., 2017; Kurtzer et al., 2017), obtaining a fraction of removed streamlines of 0.890 (0.857, 0.926) for the data.

To disentangle the effects of the number of streamlines from the tractography method, a comparison using the same number of streamlines and subjects would be necessary.

### 4.2 Agreement between rSIFT and rCOMMIT

**Figure 1:**
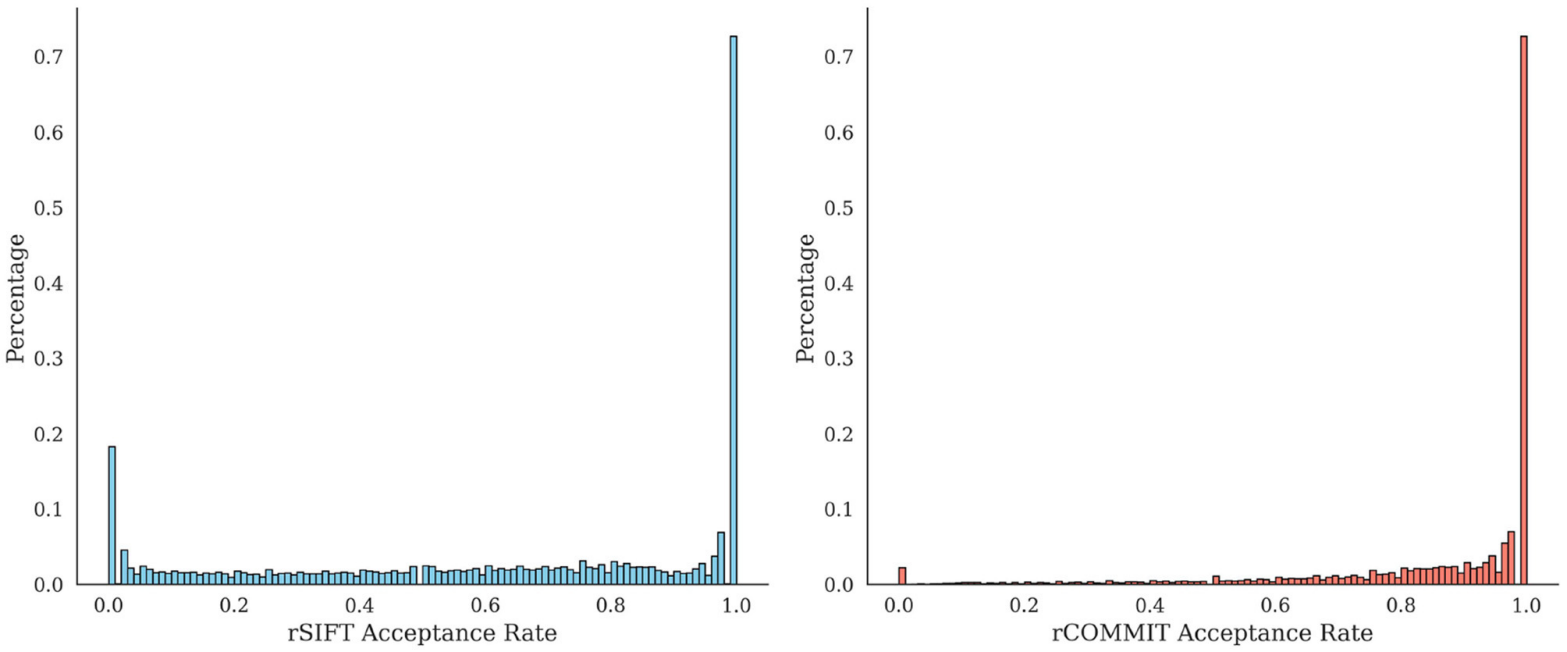
Distribution of rSIFT acceptance rates **(Left)** for accepted streamlines by rCOMMIT (acceptance rate = 1) and distribution of rCOMMIT rates for accepted streamlines by rSIFT **(Right)**. The percentages are given for the total number of streamlines from all subjects in the dataset.

**Figure 2:**
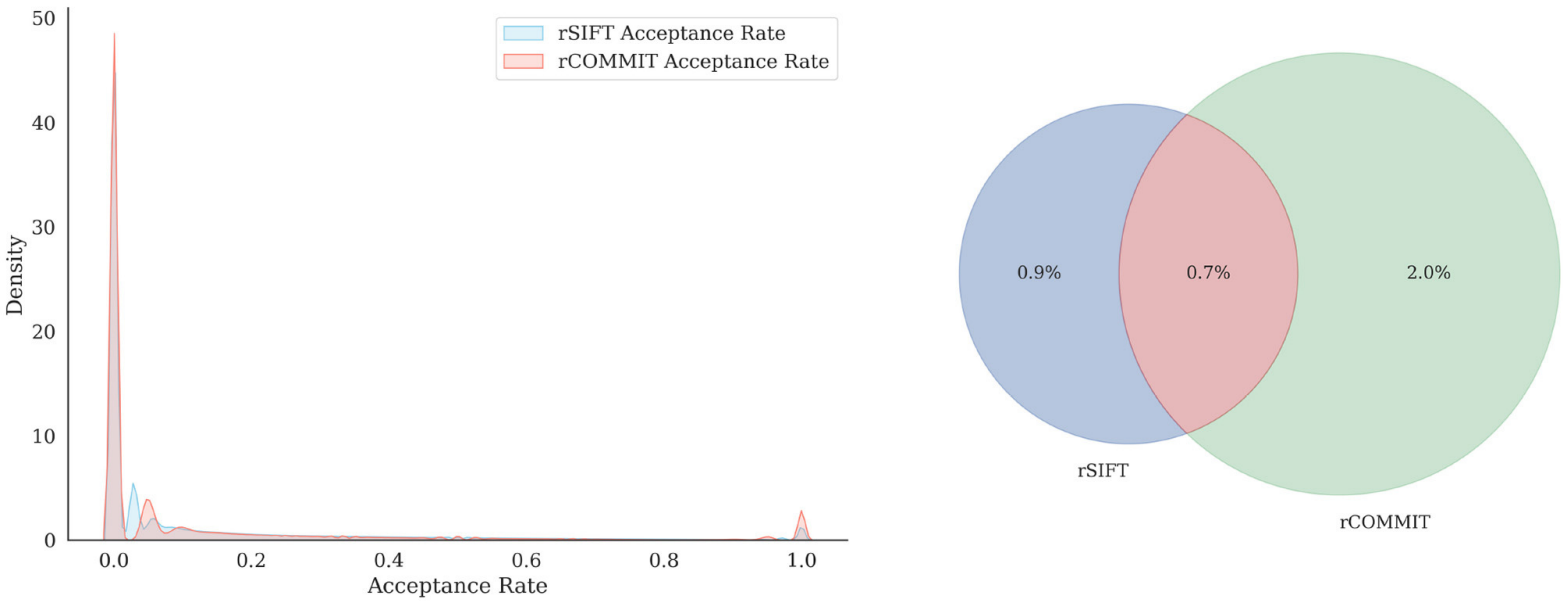
Distribution of the acceptance rate **(Left)** for rSIFT and rCOMMIT acceptance rates and the proportion of overlapping streamlines with a threshold of 1 **(Right)**.

It is interesting to assess the agreement between rCOMMIT and rSIFT for further estimations of redundancy. Figure 1 shows the distribution of rSIFT and rCOMMIT acceptance rates of streamlines that are accepted by the other method for all subjects in the HCP 10M dataset. As shown, the two methods have a large number of streamlines where both have an acceptance rate of 1.0, but there are many other streamlines where the two methods disagree. Figure 2 shows the distribution of acceptance rates and the Venn diagram between the two sets of accepted streamlines per method. As shown, the distributions have high concentrations around 0/1. Further, it should be noted that rSIFT disregards more streamlines than rCOMMIT, and the intersection of the two sets is 0.7% of the whole dataset. That means that only around 70,000 streamlines out of 10 million are always accepted by rSIFT and rCOMMIT. This observation has also been reported by Wan (2023). This suggests that using intersection or minimal acceptance rates may be too tight to estimate the upper bounds of redundancy compared to a single run of the corresponding method.

### 4.3 Upper bounds

We implement the upper bounds by sub-sampling in Section 3.3.1 using Hoeffding's inequality and the empirical Bayesian approach in Section 3.3.2 for our dataset, for which we have the rCOMMIT and rSIFT results. The bounds are computed for different estimates of the streamlines FDR given by rCOMMIT, rSIFT, maximal valid filtering and pooled filtering. We do not include the intersection of rSIFT and rCOMMIT in these experiments because it requires a threshold that is difficult to set. As discussed previously, the minimal acceptance rate is similar to the intersection and has the advantage of not needing thresholding.

**Figure 3:**
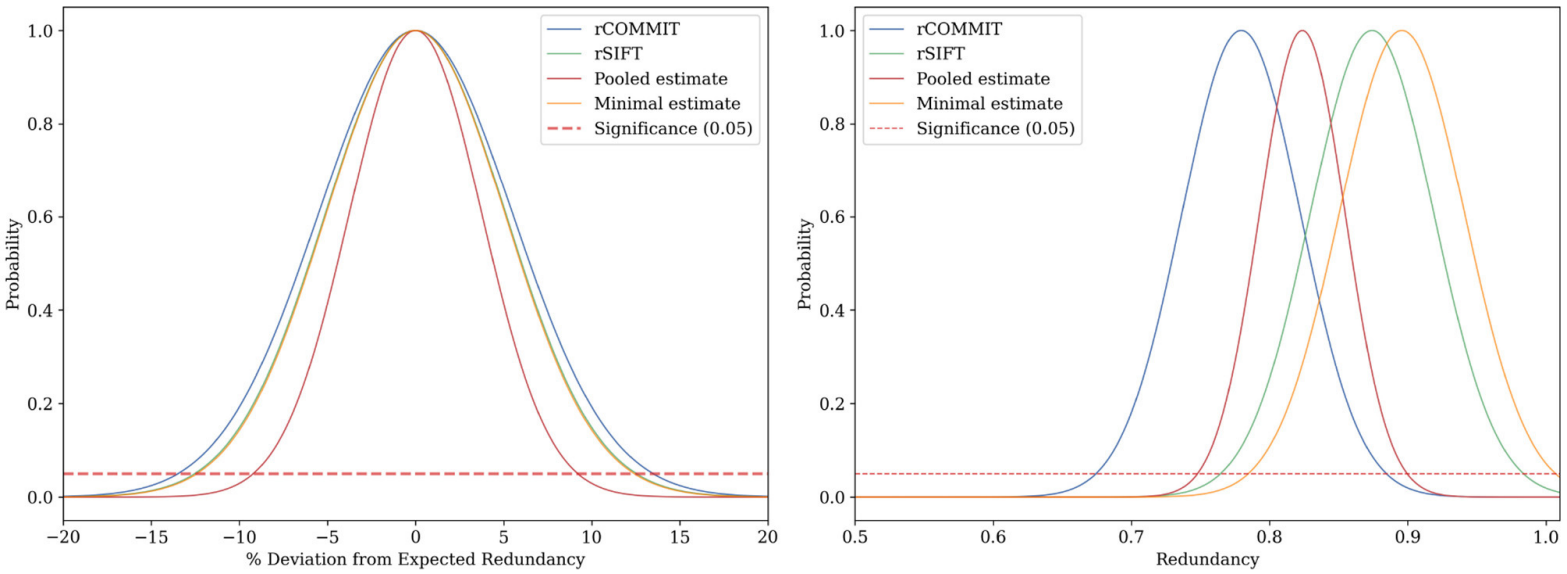
FDR estimation for subject ID 877168 in the HCP dataset using Hoeffding's inequality using different estimators of FDR. **(Left)** Deviation of the sampled FDR from its expected value. **(Right)** Hoeffding's upper-bound estimation of the FDR.

**Figure 4:**
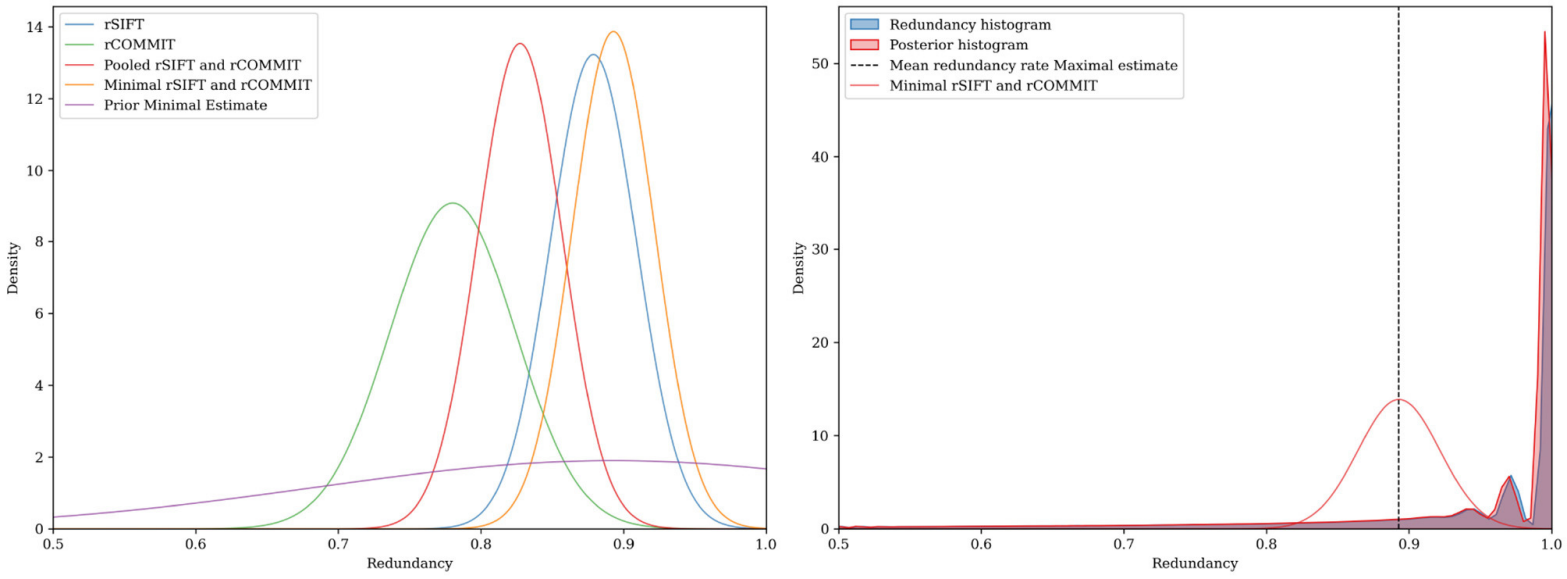
FDR upper-bound estimation using a Bayesian approach for subject ID 877168 in the HCP dataset. **(Left)** The estimate is determined by the width and the center of the distribution. **(Right)** In this example, the posterior and FDR histogram approximately coincide due to the extensive subsets for rSIFT and rCOMMIT, but generally, the posterior will be shifted in the direction of the prior for the model.

Figures 3, 4 show the results for a specific subject from our dataset. As shown, the maximal filtering approach provides the most strict upper bound of the FDR, followed by rSIFT. As was previously seen, rCOMMIT generally filters fewer streamlines than rSIFT, and we also note that the variance of rCOMMIT results is wider for the data, especially for the Bayesian approach, suggesting that COMMIT may be a less stable filtering method. The pooled estimate is approximately in the middle between rSIFT and rCOMMIT and has the lowest variance due to the combined subsets.

Table 2 shows Hoeffding's bound aggregated over all subjects in the dataset. These results are consistent with the ones in Figures 3, 4. It should be noted that the minimal estimate gives an upper bound of 1.0, suggesting that (almost) all streamlines are classified as false positives. As discussed before, the number of streamlines that are accepted by both rSIFT and rCOMMIT is very low, which makes the minimal estimate too strict. It should also be noted that Hoeffding's bound is relatively wider than the Bayesian one (compare the difference between the mean FDR and Hoeffding's bound in Table 2 to the mean posterior and upper bound in Table 3).

**Table 2:** Table of aggregated results for subjects for Hoeffding's bound ≤ 0.05 computed by Equation 2.

**FDR estimate**	**Mean FDR**	**Mean Hoeffding's bound**	**95% CI**
rSIFT	0.868	0.978	(0.971, 0.985)
rCOMMIT	0.772	0.869	(0.852, 0.886)
Pooled estimate	0.817	0.890	(0.880, 0.901)
Minimal estimate	0.899	1.000	(1.000, 1.000)

**Table 3:** Table of aggregated results for all subjects for empirical Bayesian upper bound.

**FDR estimate**	**Mean posterior**	**Mean upper bound**	**95% CI**
rSIFT	0.873	0.934	(0.929, 0.939)
rCOMMIT	0.755	0.850	(0.815, 0.885)
Pooled estimate	0.818	0.880	(0.869, 0.892)
Minimal estimate	0.890	0.948	(0.936, 0.960)

**Figure 5:**
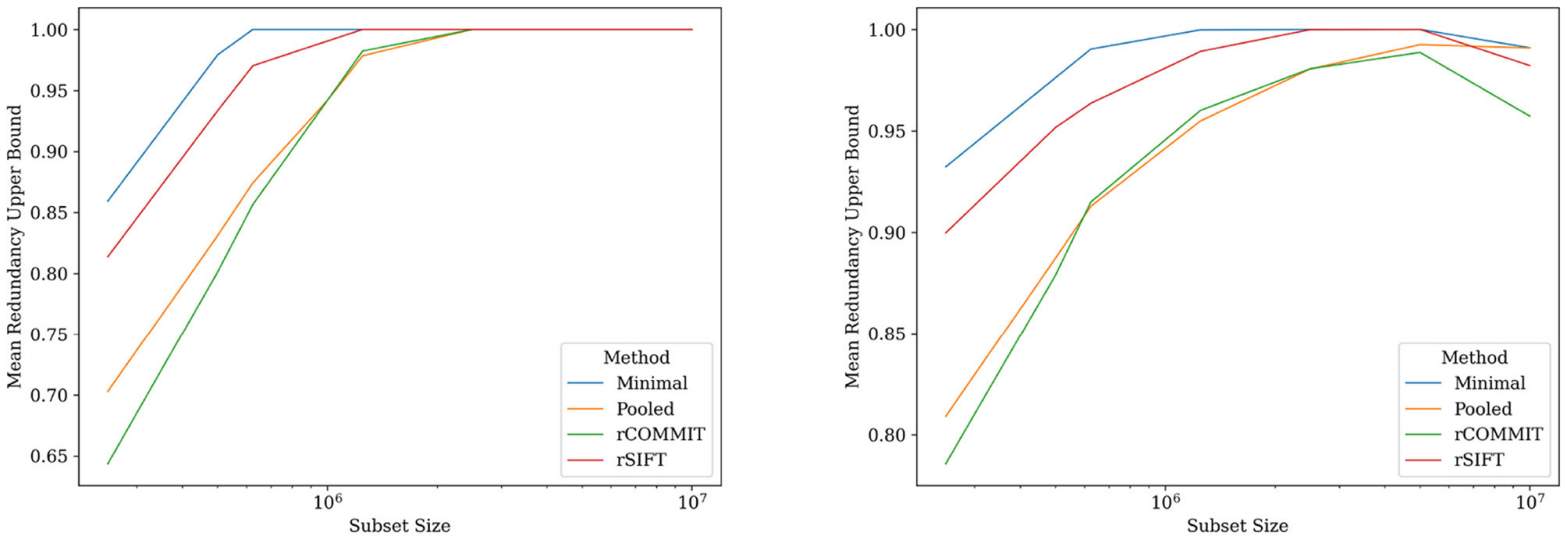
FDR upper bounds per subset size for different estimates of the streamline probabilities. **(Left)** Hoeffding's upper bound for log-normalized subset sizes for rSIFT and rCOMMIT. **(Right)** Bayesian upper bound for log-normalized subset sizes for rSIFT and rCOMMIT.

The empirical Bayesian upper bound gives a tighter upper bound with estimates and confidence intervals reported in Table 3. The Bayesian approach is less strict than Hoeffding's bound. Still, both methods show a similar trend between the probability estimators. Table 4 shows the FDR estimated with the different methods and we observed that the posterior Bayesian distribution of Table 3 approximately matches the mean FDR from Table 4. This is expected since the number of subsets of rSIFT and rCOMMIT in the dataset is significant enough to dominate the posterior probabilities, i.e., the confidence in the empirical estimate increases with the number of subsets. Figure 5 shows the relation between the subset size and the FDR for both Hoeffding's and Bayesian upper bounds.

**Table 4:** Table of aggregated results for all subjects for the average number of filtered streamlines, that is $1-\bar{a}$, which is the mean acceptance rate for all subjects.

**FDR estimate**	**Mean FDR**	**95% CI**
rSIFT	0.873	(0.866, 0.880)
rCOMMIT	0.754	(0.703, 0.805)
Pooled estimate	0.817	(0.801, 0.834)
Minimal estimate	0.896	(0.886, 0.907)

## 5 Discussion

### 5.1 Effectiveness of bounds

The effectiveness of our bounds is underscored by their capacity to accommodate the inherent variability of tractography data. In our dataset, we predict an FDR that is bounded between 85.7% [the lower confidence of interval (CI) of the ExTractor method] and 96% as given by the upper bound of the confidence interval of the minimal estimate for the empirical Bayesian method in Table 3. This interval includes all estimated upper bounds by our proposed methods except for the minimal estimate with Hoeffding's bound, which we conclude gives a too-strict upper bound. These results suggest that the redundancy consists of at least 10% of streamlines (1 million), excluding those that are also anatomically implausible, i.e., the difference between the lower and upper bounds.

Our lower bound, derived from the ExTractor algorithm, confirms the presence of anatomically implausible streamlines, providing a foundation upon which redundancy can be objectively assessed. The upper bounds, constrained by Hoeffding's inequality and the empirical Bayesian approach, provide different lenses through which the tractography-filtering outcomes can be evaluated. These statistical methods offer both means to assess the redundancy and also serve as means to understand the differences between different tractography filtering approaches. The variation in the results between these upper-bound methods reveals the trade-offs between non-parametric results and the incorporation of prior knowledge into streamlines' FDR estimation. We suggest three methods for combining the tractography filtering results from rSIFT and rCOMMIT for the upper-bound computations. The intersection between rSIFT and rCOMMIT determines the streamline's acceptance by thresholding the results from both tractography filtering algorithms. The strategy of pooling combines the streamlined acceptance of both methods to decrease the uncertainty and bias toward any particular method. The minimum acceptance rate strategy uses a subset-level approach to determine the intersectional streamline acceptance rate as the minimum of each method.

### 5.2 Difference between upper-bound methods

The two different statistical approaches for bounding the redundancy of the tractogram give similar results but are based on different assumptions based on the data. Hoeffding's inequality, as a non-parametric method, does not make assumptions about the distribution of the streamline false discovery rate. Its bounds are generally less tight than those of the Bayesian method but cover a broader range of potential tractography scenarios. Meanwhile, the empirical Bayesian approach offers a different perspective by introducing prior knowledge into the analysis, narrowing down the potential variance in tractogram redundancy. The results of this method rely on the prior chosen to represent the initial distribution of the data, and in cases of limited data, the effect of the prior will be enhanced, and a poorly chosen prior could lead to misleading conclusions.

When comparing Hoeffding's inequality and the empirical Bayesian approach, the perspective from which they view the data is different. Hoeffding's inequality assesses the redundancy of tractograms at the subset level. It treats each subset as an independent event, and the focus is on the resulting aggregate of these subsets. It does not delve into the individual characteristics of streamlines but instead evaluates the larger pattern of redundancy across the entire collection of subsets. This approach is particularly useful in providing a high-level, macroscopic understanding of the redundancy.

In contrast, the empirical Bayesian approach considers the evidence for each streamline on an individual basis. This perspective allows it to incorporate prior knowledge specific to each streamline's behavior across different subsets. By looking at the streamline acceptance rates, the empirical Bayesian approach effectively combines evidence from multiple iterations to update the prior beliefs into a posterior distribution reflective of each streamline's probability of being redundant. This approach values the individual contribution of streamlines within the tractogram. Figure 5 further shows the difference between the upper-bounding methods on different subsets, visualizing the interplay between the Bayesian empirical model and the data for each subset. The reduction in the Bayesian estimate (Figure 5, right) for the entire tractogram is due to the low variance of SIFT and COMMIT when run on a single fixed tractogram.

### 5.3 Efficiency of redundancy estimation methods

Many tractogram filtering methods are based on the exact composition of the tractogram (Smith et al., 2013, 2015; Daducci et al., 2015; Schiavi et al., 2020) and fail to take into account the uncertainty inherent to not only probabilistic tractography methods but the DW-MRI signal. Randomized SIFT and COMMIT (Hain et al., 2023; Wan, 2023) are methods that aim to assess the stability of their underlying filtering method, but due to computational time, these have challenges with widespread utilization. It should be noted that ExTractor (Petit et al., 2023) is also very expensive.

Machine learning approaches are promising to reduce the burden of computations. For example, Astolfi et al. (2023), Hain et al. (2023), Wan (2023), and (see text footnote ^1^) used deep learning for approximate ExTractor, rSIFT and rCOMMIT, respectively. We decided not to use the method by Astolfi et al. (2023) to obtain more accurate estimations of the lower bound of the tractogram FDR. As for rSIFT and rCOMMIT, the deep learning methods aim to classify individual streamlines from the streamline coordinates. Thus, duplicates will inexorably be accepted by the neural networks, making them inappropriate for estimating the upper bound, although they could potentially be used for estimating the lower bound provided that their accuracy is good enough.

We chose ExTractor to estimate the lower bound since it is based on neuroanatomical knowledge, making it more closely related to assessing anatomical plausibility. As discussed by Petit et al. (2023), ExTractor still can have problems with false negatives, which can affect the estimation of the lower bound of the FDR. That might imply that the lower bound estimated with ExTractor might become too strict. While FINTA (Legarreta et al., 2021) might be a good alternative to ExTractor for estimating the lower bound of redundancy because of its speed, it lacks explainability. Moreover, FINTA requires setting thresholds per bundle that are difficult to generalize for whole-brain tractogram filtering. Indeed, more research is needed to address the current issues with these methods.

### 5.4 Weight-based tractogram filtering methods

Some tractogram filtering methods produce a weight for each streamline that reflects its contribution to the diffusion signal. Examples of these methods are COMMIT (Daducci et al., 2015), SIFT2 (Smith et al., 2015), and COMMIT2 (Schiavi et al., 2020). Effectively, this means that streamlines with a weight of 0 are removed from the tractogram. SIFT2 (Smith et al., 2015) is motivated by the computational inefficiency of generating highly redundant tractograms and proposes to estimate an effective area of each streamline. The result is a weight for each streamline that can be used to compute a post-filtering weighted tractogram without removing streamlines unless the weight is zero. A limitation to this approach is that there is no explicit removal of anatomically implausible streamlines unless the weight is zero, leading to a greater emphasis on an accurate original tractogram.

Building upon the efforts to increase the anatomical accuracy of tractography, Schiavi et al. (2020) introduced COMMIT2, a refinement of the original COMMIT framework. COMMIT2 enhances the specificity of reconstructing brain networks by considering their organization into anatomically plausible bundles. By balancing the local axon density derived from the diffusion-weighted MR signals against the sparsity of bundles used to explain that density, COMMIT2 suppresses the number of false positive connections more effectively compared to COMMIT, SIFT, and SIFT2, possibly at the cost of sensitivity.

As discussed in Jörgens et al. (2021), the scores of SIFT2 as compared to SIFT are not directly related to redundancy. That is, in a sample of a tractogram, a streamline can be disproportionately highly weighted compared to its significance in another sample of the same tractogram since individual streamline weights are determined by the other streamlines.

Since the inputs of the upper-bound estimations are estimations of redundancy (acceptance rates) per streamline, weights from such tractography filtering methods cannot be used directly for our purposes. That problem can be solved by a method that can estimate the probability of acceptance from those scores. Proposing such a method is part of our current research.

### 5.5 Application area

The proposed methods for bounding tractogram redundancy have implications for selecting tractography methods, optimizing the number of streamlines, and choosing filtering algorithms. The capability to quantify redundancy makes it possible to systematically compare the efficacy of different tractography approaches, understand how each method contributes to redundancy in the tractograms they produce, and possibly improve the methods. This quantification can guide the selection of tractography algorithms that balance the requirements of completeness and efficiency.

Additionally, measuring the effect of the number of streamlines on the overall redundancy is a potential application for the established bounds. Streamline counts can be adjusted based on empirical evidence of redundancy, facilitating the configuration of tractography pipelines to produce tractograms that are both informative and resource-efficient. The methods for bounding redundancy could also assist in evaluating the performance of various tractography filtering algorithms with a quantitative metric. Such evaluations can determine how different filtering methods reduce redundancy and enhance the anatomical plausibility of tractograms. These measurements can be used to complement the traditional tractometer measurements (Côté et al., 2013). Tractometry methods are also highly dependent on a high-quality tractogram, and our study contributes to the area of being able to measure tractogram quality and fidelity, starting with redundancy.

The introduced method can be further developed to benchmark both tractograms and filtering algorithms to assess the stability of their results as well as the underlying tractogram redundancy.

### 5.6 Limitations

Both Hoeffding's inequality and the empirical Bayesian approach offer valuable frameworks for estimating the FDR; however, they do not account for the variable topographical complexity of brain regions. The current methods treat the tractogram as a homogeneous entity and apply a uniform standard across all regions, potentially overlooking these variations.

Furthermore, our approach does not incorporate region-specific biological knowledge about white matter pathways that could significantly inform the process of identifying redundancy. Instead, it relies on the underlying tractogram filtering methods—ExTractor, rSIFT, and rCOMMIT—to give appropriate estimates of streamline-level redundancy.

ExTractor provides a rule-based approach to filter anatomically implausible streamlines, but the definition of anatomical plausibility is not unequivocal; therefore, any rule-based approach may filter connections that are truly positive and miss erroneous streamlines. We recognize this limitation and use the ExTractor as a method to estimate the proportion of geometrically plausible streamlines. It is, however, an approximation for a lower bound that excludes implausible streamlines but does not optimize the representation of the underlying diffusion signal.

The computational load of establishing these bounds is currently significant; for example, processing each subject with rCOMMIT takes ~2 weeks on a high-performance workstation with a 16-core Intel Xeon processor and 64 GB of RAM. We, however, note that the acceptance probabilities for each streamline, as given by rSIFT and rCOMMIT in Table 4, are indicative of the posterior distribution of each streamline. We, therefore, argue that approximating the streamline acceptance rate with a noise-injected deep learning model could be an interesting alternative to explore to establish the confidence interval over sampled outputs from the models. This approach would provide a practical estimate of the redundancy, and previous research has shown this to be a feasible route (Legarreta et al., 2021; Astolfi et al., 2023; Hain et al., 2023; Wan, 2023) (see text footnote ^1^). While this is true for estimating the lower bound of the false discovery rate, training a model that can be used for estimating the upper bound of the FDR is more challenging and deserves additional research.

### 5.7 Future studies

The proposed method for statistically bounding redundancy in a tractogram offers several potential future areas of research. Our study is based on computational methods for filtering tractograms, and comparing our results to histological data could provide a prior for the expected redundancy, similar to what has been done for the tractogram fidelity (Seehaus et al., 2013; Delettre et al., 2019).

**Figure 6:**
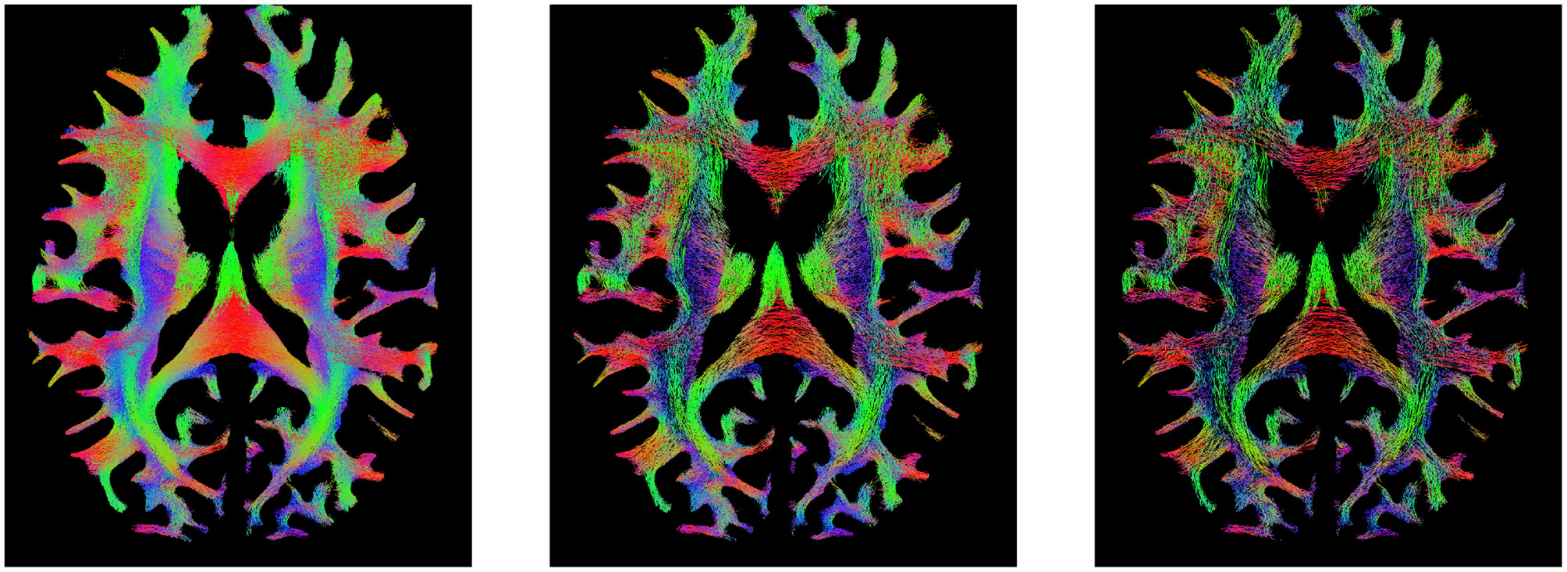
Visualization of the tractogram of Subject 877168 in HCP 10M. **(Left)** Tractogram filtering by rCOMMIT. **(Middle)** Tractogram filtering by rSIFT. **(Right)** Foundational streamlines at the intersection of filtering by rCOMMIT and rSIFT with acceptance probability = 1.

In the intersection of rCOMMIT and rSIFT, we find that there are certain streamlines that build the overarching structure of the tractogram that appear uniformly over the entire tractogram (see Figure 6). We denote them *foundational streamlines*, and these may be suggested to form the basis for the tractogram. In this study, we do not investigate whether these streamlines retain individual characteristics or whether tractography filtering can compress a tractogram.

Furthermore, in our study, we apply our methods with rSIFT and rCOMMIT, but there have been studies extending these methods, such as SIFT2 (Smith et al., 2015), COMMIT2 (Schiavi et al., 2020), and the blurred streamlines representation in combination with COMMIT proposed by Gabusi et al. (2024). Extending our proposed redundancy metric to these methods is an important avenue for our future research. Regarding SIFT2, the weights estimated by the method are always positive, according to Jörgens et al. (2021), which makes it impossible to use the same randomization procedure we use for SIFT and COMMIT to SIFT2. In turn, COMMIT, and consequently COMMIT2, encourages sparsity on the weights. As a consequence, COMMIT2 can give zero weight to many streamlines, making it suitable for our randomization procedure. The same is true for other approaches based on COMMIT (e.g., COMMIT-tree Ocampo-Pineda et al., 2021 or COMMIT-T2 Barakovic et al., 2021). Similarly, assessing the redundancy of clinical datasets could provide further insights into the variation depending on the diffusion MRI quality. For this, it is relevant to study randomized methods that can run on single-shell diffusion data.

## 6 Conclusion

We have presented two statistical approaches for bounding the redundancy with minimal assumptions that can be applied to different tractography filtering methods, with examples given for the randomized SIFT and COMMIT. Our approaches are designed to be applicable across a variety of filtering methods and offer reliability in heterogeneous datasets. While there remain areas for further validation, the methods developed comprise a step forward toward quantifying the lower and upper bounds of the false discovery rates of streamlines in tractograms and the redundancy rate and can provide a viable metric for the quality of tractography methods. Future research includes evaluating the proposed bounds on different tractography methods and aims toward ranking tractography methods by their redundancy to give an application-dependent recommendation of the number of streamlines that are necessary for a good representation of brain neural tracts.

## Data availability statement

Publicly available datasets were analyzed in this study. This data can be found here: https://db.humanconnectome.org.

## Ethics statement

Ethical review and approval was not required for the study on human participants in accordance with the local legislation and institutional requirements. The participants provided their written informed consent to participate in this study.

## Author contributions

SP: Conceptualization, Investigation, Methodology, Software, Validation, Visualization, Writing – original draft, Writing – review & editing, Formal analysis. RM: Conceptualization, Funding acquisition, Methodology, Project administration, Resources, Supervision, Visualization, Writing – review & editing.

## Footnotes

^1^Persson, S., Wan, X., and Moreno, R. (Submitted). *Randomly Committing: Iterative Convex Optimization for Microstructure-Informed Tractography*.
